# Biliary Drainage for the Preoperative Management of Periampullary Neoplasms: A Retrospective Cohort Study

**DOI:** 10.3390/medicina61091565

**Published:** 2025-08-30

**Authors:** Septimiu A. Moldovan, Emil I. Moiș, Florin Graur, Vlad I. Nechita, Luminiţa Furcea, Florin Zaharie, Raluca Bodea, Simona Mirel, Mihaela Ș. Moldovan, Tudor Mocan, Zeno Spârchez, Andrada Seicean, Nadim Al Hajjar

**Affiliations:** 1 Department of Surgery, “Octavian Fodor” Regional Institute of Gastroenterology and Hepatology, Croitorilor Str., No. 19–21, 400162 Cluj-Napoca, Romania; septimiu1995@yahoo.com (S.A.M.); graurf@yahoo.com (F.G.); nechita.vlad@umfcluj.ro (V.I.N.); luminita.furcea@yahoo.com (L.F.); florinzaharie@yahoo.com (F.Z.); ralucabodea@yahoo.com (R.B.); na_hajjar@yahoo.com (N.A.H.); 2 Department of Surgery, “Iuliu Hațieganu” University of Medicine and Pharmacy, Croitorilor Str., No. 19–21, 400162 Cluj-Napoca, Romania; 3 Department of Medical Informatics and Biostatistics, “Iuliu Hațieganu” University of Medicine and Pharmacy, Louis Pasteur Str., No. 6, 400349 Cluj-Napoca, Romania; 4 Department of Medical Devices, “Iuliu Hațieganu” University of Medicine and Pharmacy, Louis Pasteur Str., No. 4, 400349 Cluj-Napoca, Romania; smirel@umfcluj.ro; 5 Department of Endocrinology, County Emergency Hospital, Endocrinology Clinic, Louis Pasteur Str., No. 3-5, 400349 Cluj-Napoca, Romania; mihaelamirel@gmail.com; 6 3rd Medical Department, “Iuliu Hațieganu” University of Medicine and Pharmacy, 400162 Cluj-Napoca, Romania; mocan_tudor@yahoo.com; 7 UBBMed Department, Babeș-Bolyai University, 400349 Cluj-Napoca, Romania; 8 Department of Gastroenterology, “Octavian Fodor” Regional Institute of Gastroenterology and Hepatology, Croitorilor Str., No. 19–21, 400162 Cluj-Napoca, Romania; zsparchez@yahoo.co.uk (Z.S.); andradaseicean@yahoo.com (A.S.); 9 Department of Gastroenterology, “Iuliu Hațieganu” University of Medicine and Pharmacy, Croitorilor Str., No. 19–21, 400162 Cluj-Napoca, Romania

**Keywords:** periampullary neoplasms, preoperative biliary drainage, straight surgery, endoscopic drainage, surgical drainage, postprocedural complications, postoperative complications

## Abstract

*Background and Objectives:* Preoperative biliary drainage (PBD) in patients with periampullary neoplasms remains a debated topic, with various techniques available and conflicting evidence regarding their impact on postoperative outcomes. This study aimed to assess, in a high-volume pancreatic surgery center, whether the choice among endoscopic, surgical, or no preoperative biliary drainage influences postprocedural and postoperative complication rates. *Materials and Methods:* A retrospective cohort study was conducted at the Surgical Department of the “Octavian Fodor” Regional Institute of Gastroenterology and Hepatology in Cluj-Napoca, Romania, between January 2017 and May 2023. A total of 655 patients undergoing pancreaticoduodenectomy or total pancreatectomy for resectable periampullary tumors were divided into three groups: no PBD, endoscopic PBD, and surgical PBD. Clinical, procedural, and postoperative variables were collected and statistically analyzed. *Results:* Endoscopic drainage was associated with a significantly higher rate of postoperative intra-abdominal abscesses, postoperative pancreatic fistula (POPF), and pancreaticojejunostomy fistula compared to surgical drainage and no PBD. Patients in the endoscopic group also exhibited significantly higher rates of positive bile cultures, particularly with pluribacterial populations. Procedure-related complications, such as pancreatitis and cholangitis, were significantly lower in the surgical drainage group. No significant differences were found among groups regarding postoperative hospital stay, relaparotomy rates, or 90-day mortality. *Conclusions:* Surgical biliary drainage was associated with lower perioperative morbidity compared to endoscopic drainage. While endoscopic drainage remains the most commonly used approach, surgical drainage may offer a safer alternative in selected patients. Prospective randomized controlled trials are warranted to validate these findings.

## 1. Introduction

Periampullary tumors represent a heterogenous class of neoplasms that arise from the pancreatic head, distal main bile duct, duodenal wall, and ampulla of Vater [1]. Their localization in the proximity of the main bile duct is associated frequently with malignant obstructive jaundice, which can lead to complications such as cholangitis, sepsis, coagulopathy, and impaired liver or kidney function [2]. If complications have emerged, straight surgery can be associated with high morbidity [3].

Ampullary tumors represent a distinct subset of periampullary neoplasms, accounting for approximately 0.6–0.8% of digestive malignancies, and are characterized by unique clinical and epidemiological features compared with pancreatic or distal bile duct cancers. They often present earlier in the disease course due to obstruction of the ampulla, which typically results in symptomatic jaundice at smaller tumor sizes, facilitating earlier diagnosis and potentially better resectability rates. Furthermore, ampullary cancers are associated with a more favorable prognosis than pancreatic adenocarcinoma, although outcomes vary significantly depending on histological subtype (intestinal versus pancreatobiliary) [4]. According to ESMO guidelines, surgical resection by pancreaticoduodenectomy is the standard treatment for localized ampullary and pancreatic tumors, but unlike pancreatic cancer, neoadjuvant therapy is not routinely indicated in ampullary tumors, and most patients proceed directly to surgery [5].

PBD aims to alleviate biliary obstruction by relieving jaundice, thereby improving liver function and reducing the risk of postoperative complications, such as infections and liver failure. The decision to perform PBD, however, depends on various factors, including the severity of jaundice, the potential for surgical resection, and the patient’s overall health status [6].

The ESGE and ASGE guidelines advise carrying out PBD in patients who present with cholangitis, where there is a delay of curative surgery for different reasons, or for those with indication of neoadjuvant oncological treatment. According to the ESMO guidelines for pancreatic cancer from 2023, a baseline bilirubin level of 250 umol/L (14.6 mg/dL) is generally considered a threshold for recommending preoperative biliary drainage. However, in specific clinical scenarios—such as the presence of cholangitis, the need for delayed surgery, or planned neoadjuvant treatment—preoperative biliary drainage may be more readily justified. Ultimately, the decision to proceed with drainage at this bilirubin level should be individualized based on the patient’s clinical context and the treating physician’s judgment [7,8].

Various techniques are available for biliary drainage, including ERCP-guided stenting, PTBD, and surgical biliary diversion. Each method comes with distinct advantages and limitations. ERCP, the most used technique, provides both diagnostic and therapeutic benefits, allowing for the placement of stents to bypass the obstructed bile duct. PTBD, on the other hand, is an alternative for patients who are not candidates for ERCP or who have more complex anatomical considerations [9].

In the preoperative management of periampullary tumors complicated by obstructive jaundice, alternative biliary drainage methods such as surgical biliary drainage and PTBD are employed when endoscopic access is not feasible or has failed. Surgical biliary drainage provides durable decompression and allows intraoperative assessment of other abdominal pathology, but its invasive nature is associated with increased perioperative morbidity, longer hospital stay, and delayed recovery. PTBD, while less invasive and suitable for patients with proximal biliary obstruction or poor surgical candidacy, may cause significant patient discomfort due to the presence of external catheters, and it carries risks such as bile leaks, hemorrhage, and tumor seeding along the drainage tract. The decision between these approaches is influenced by anatomical complexity, patient comorbidities, clinical urgency, and the experience of the treating center.

Surgical drainage of the common bile duct may be achieved through biliodigestive diversion, cholecystostomy, or the insertion of a Kehr tube. Whenever feasible, a laparoscopic or robotic approach is preferred over open surgery, given its advantages in reducing postoperative morbidity and promoting faster recovery [10].

Although choledochoduodenostomy was historically a cornerstone in the management of biliary obstruction due to its effectiveness in decompressing a dilated common bile duct, its role has markedly diminished in the modern era. The widespread availability and success of ERCP and laparoscopic bile duct exploration have largely supplanted the need for this once-routine surgical procedure. Nonetheless, choledochoduodenostomy remains a valuable salvage option in cases of failed or repeatedly unsuccessful ERCP procedures [11].

Hepaticojejunostomy is often favored in malignant cases, as tumor progression may compromise duodenal anastomosis through dehiscence or obstruction. A Roux-en-Y configuration is typically employed to minimize the risk of cholangitis by preventing enteric reflux into the biliary system [12].

This introduction explores the various techniques of preoperative biliary drainage, their indications, and the potential benefits and risks in the context of periampullary tumors. Understanding these options is crucial for optimizing patient care and guiding clinical decision-making to achieve the best possible outcomes for these challenging cases.

While PBD has been widely used to improve perioperative outcomes in patients with resectable periampullary tumors, its true benefits remain controversial. Previous studies and meta-analyses have reported conflicting results, with some suggesting reduced postoperative complications and others highlighting increased risks of infection, bile contamination, and stent-related morbidity. Recent studies suggest that PBD may not always confer survival benefits in patients who are candidates for curative resection. Moreover, the optimal approach—whether endoscopic, surgical, or no drainage at all—remains uncertain, particularly in high-volume pancreatic surgery centers.

The present study aims to clarify this ongoing debate by analyzing a large single-center cohort of 655 patients who underwent curative pancreatic resection. We specifically investigated whether the choice of drainage method (endoscopic, surgical, or none) influenced postprocedural and postoperative complication rates. By directly comparing outcomes among these groups, our work provides novel evidence of the safety and efficacy of surgical drainage as an alternative to endoscopic stenting, with implications for clinical decision-making and guideline development.

## 2. Materials and Methods

### 2.1. Study Design

The design of our study consisted of a monocentric retrospective cohort study, undertaken at the Surgical Department of Regional Institute of Gastroenterology and Hepatology “Prof. Dr. Octavian Fodor”, Cluj-Napoca, Romania, between 1 January 2017 and 31 May 2023.

Inclusion criteria consisted of all patients admitted between January 2017 and May 2023 to the Surgical Department of the “Octavian Fodor” Regional Institute of Gastroenterology and Hepatology, Cluj-Napoca, with a diagnosis of resectable periampullary tumors (benign or malignant) who underwent surgical treatment with curative intent. Eligible surgical procedures were pancreaticoduodenectomy (classic Whipple or pylorus-preserving technique) and total pancreatectomy. Patients were further categorized according to the type of preoperative biliary drainage (PBD) strategy applied: no drainage, endoscopic drainage (ERCP with plastic or self-expandable metallic stents), or surgical drainage (cholecystostomy, T-tube insertion in the common bile duct, or biliodigestive diversion such as choledochoduodenostomy or hepaticojejunostomy).

Exclusion criteria were patients who underwent percutaneous biliary drainage (PTBD—seven patients or percutaneous cholecystostomy—one patient), as their small number (n = 8) precluded meaningful statistical comparison. Patients who were not eligible for surgery with curative intent, including those with unresectable disease at presentation, disease progression prior to surgery, or drainage-related complications preventing resection, were also excluded from the analysis.

We enrolled 655 patients who presented at our service with resectable periampullary tumors. Pancreaticoduodenectomy was performed in 599 patients, while 56 patients benefited from total pancreatectomy. The decision to perform a total pancreatectomy was made intra-operatively by the surgeon, taking into consideration the extent of the tumor, the consistency of the pancreas, and the presence of preoperative diabetes. The aim of our study was to establish which PBD method confers a better perioperative outcome. For that reason, we constructed three groups of patients based on PBD technique, with 276 patients having no prior PBD, 327 patients with endoscopic drainage, and 52 patients with surgical drainage. The surgical approach was performed either by laparosopy or laparotomy.

Furthermore, it is clinically relevant that 38 patients in the endoscopic group required re-stenting (31 patients once, 7 patients twice).

The decision to perform PBD was based on multidisciplinary team discussions involving gastroenterologists, anesthesiologists, radiologists, oncologists, and surgeons, with the final decision made by the attending hepatobiliopancreatic surgeon, in accordance with our institutional protocol. Selection of patients for endoscopic or surgical biliary drainage was not determined by a fixed bilirubin cut-off, but rather individualized according to clinical status, imaging findings, and multidisciplinary assessment. Baseline bilirubin level was not used as an independent criterion for assigning patients to a specific management strategy and was therefore not uniformly recorded across the three groups. In line with our center’s standard of care, patients eligible for radical resection were managed preoperatively under the supervision of the surgical team, which retained primary responsibility for the therapeutic strategy.

When antibiotic prophylaxis was indicated before ERCP, cefazolin (2 g) or ceftriaxone (1 g) was used. In line with ESGE guidance, routine prophylaxis before ERCP was not employed, but it was administered in cases of anticipated incomplete biliary drainage, in severely immunocompromised patients, or when performing cholangioscopy [13]. In patients presenting with cholangitis, empiric antimicrobial therapy was initiated based on severity and anticipated bacterial spectrum.

In practice, ERBD was preferred whenever endoscopic expertise and immediate access to ERCP were available, especially in patients without prior upper gastrointestinal surgery and with favorable anatomy. Conversely, surgical drainage was selected in situations where endoscopic or percutaneous access was not feasible or available, in cases of failed ERBD, or when patients required an open abdominal procedure for another indication (e.g., exploratory laparotomy, gallbladder removal, or bypass for gastric outlet obstruction).

In cases where endoscopic biliary drainage was not feasible or failed, the choice between PTBD and surgical biliary drainage was made through multidisciplinary team discussions. Surgical drainage was favored in patients scheduled for exploratory laparotomy or when additional surgical procedures (e.g., bypass) were anticipated, whereas PTBD was reserved for patients with complex main biliary duct obstructions or those not immediately eligible for surgery.

Importantly, in patients without clear indications for preoperative biliary drainage—such as those with moderate, asymptomatic jaundice and no evidence of cholangitis or delayed surgery—no PBD was performed, in line with current NCCN and ESMO guideline recommendations.

Final decision-making adhered to institutional protocols, with the hepatobiliopancreatic surgeon maintaining primary responsibility for preoperative management strategy.

This study adheres to the STROBE guidelines, with the STROBE checklist attached to the Supplementary Materials.

### 2.2. Data Collection

Patients’ characteristics were collected, alongside surgical treatment and tumoral histology, from our center’s electronic database. PD was performed as either a classic Whipple procedure or by pylorus preserving technique (PPPD). Vascular invasion, when present, was resolved by tangential resection and suturing or by resection and termino-terminal anastomosis. The anastomosis of the pancreas was obtained by either PJ in a duct-to-mucosa fashion or by PG by invagination. Intraoperative parameters included blood loss and operative time.

The primary endpoint of this study was the overall postoperative complication rate, defined as the occurrence of at least one adverse event during the postoperative course following pancreatic resection. The secondary endpoints included the following: (i) procedure-related complications associated with PBD (pancreatitis, cholangitis, hemorrhage, perforation, biliary leak, and duration of drainage); (ii) specific postoperative complications such as wound infection, intra-abdominal abscess, sepsis, PPH, lymph leakage, POPF (and its subtypes depending on the pancreatic anastomosis—PJ, PG, pancreatic stump), biliary leakage, gastrojejunostomy leakage, DGE, celiac axis ischemia, mesenteric infarction, pulmonary complications, cardiovascular complications, *Clostridium difficile* infection, and MOF; (iii) microbiological findings from intraoperative bile cultures (negative, monobacterial or pluribacterial); and (iv) prognostic outcomes, including relaparotomy rate, postoperative hospital stay, and 90-day mortality.

For the purposes of analysis, baseline characteristics and postoperative outcomes were compared across all three study groups (no PBD, endoscopic drainage, and surgical drainage). In contrast, procedure-related complications (such as pancreatitis, cholangitis, perforation, hemorrhage, biliary leak, and duration of drainage) were analyzed only between the two groups that underwent a PBD procedure (endoscopic or surgical), as such events cannot occur in patients who proceeded directly to surgery without drainage.

### 2.3. Outcomes and Definitions

According to the Cotton criteria and the revised Atlanta classification, post-ERCP pancreatitis was defined as new or worsened abdominal pain that occurred within 24 h of the procedure, that required hospitalization or an extension of planned admission, and was accompanied by an elevation of serum lipase and/or amylase to at least three times the upper limit of normal [14,15]. Cholangitis is defined as the onset of fever (≥38 °C) and clinical signs of systemic inflammation (such as chills and leukocytosis) 24 to 72 h after ERCP, along with biochemical evidence of cholestasis (elevated bilirubin and/or alkaline phosphatase) and no other discernible source of infection, according to the 2018 Tokyo Guidelines modified for the post-procedural context [16]. According to ASGE guidelines, a perforation is defined as an aberrant connection between the gastrointestinal tract and surrounding spaces that can be verified by endoscopic observation, imaging results, clinical symptoms, or the necessity for radiological or surgical intervention [17]. According to ASGE standards, post-ERCP hemorrhage was described as bleeding that occurred during or after ERCP, usually during sphincterotomy, and was indicated by gastrointestinal bleeding symptoms, a hemoglobin reduction of ≥2 g/dL, or the requirement for hemostatic or transfusion intervention [17,18].

The percentage of patients who experienced at least one postoperative adverse event, regardless of its kind or severity, over the specified follow-up time was known as the overall complication rate. The following infectious complications were examined: sepsis (a systemic inflammatory response to infection, characterized by fever or hypothermia, leukocytosis or leukopenia, and signs of organ dysfunction); intra-abdominal abscess (localized fluid collection with signs of infection confirmed by imaging or intraoperatively); and wound infections (erythema, purulent drainage, or positive wound cultures requiring treatment) [19]. Any bleeding that occurs following pancreatic resection, regardless of the time, location, or intensity, is considered PPH, according to the general criteria established by the ISGPS [20]. The presence of lymphatic fluid in postoperative abdominal drainage, which is identified by its milky or opalescent appearance and a triglyceride concentration of at least 110 mg/dL (1.2 mmol/L), is known as a chyle leak [21]. According to ISGLS standards, bilious drain output with a bilirubin content at least three times more than serum levels was considered a postoperative biliary fistula [22]. ISGPS defined a POPF as drain fluid that had an amylase level on or after postoperative day 3 that was more than three times the upper limit of normal serum amylase and linked to a clinical impact (e.g., infection, need for intervention, or prolonged drainage). According to ISGPS criteria, DGE is defined as the inability to restart a solid diet within a predetermined postoperative timeframe or the inability to tolerate oral intake because of reduced stomach motility, necessitating the ongoing use of a nasogastric tube [23].

### 2.4. Statistical Analysis

Data was analyzed using R Commander 4.0.5 (R Foundation for Statistical Computing, Vienna, Austria). For qualitative data, the Chi-square test or Fisher exact test were used to evaluate the difference in frequencies between the groups. For quantitative data, comparisons between groups were conducted in two forms: parametric (Student-T test) or non-parametric tests (Mann–Whitney U) for independent groups. When more than three groups were compared, one-way ANOVA was conducted for normal distribution and Kruskal–Wallis was used when normal distribution was not present. Normality of quantitative data analyzed was assessed by the Shapiro–Wilk test for small sample sizes (n < 50) or the Kolmogorov–Smirnov test for large sample sizes (n > 50). Normality was assessed also by the Anderson Darling test for better sensitivity. In the case of normally distributed data, equality of variances was tested by Levene’s or Barlett’s tests. For equal variances, Fischer’s ANOVA was used, while for unequal variances, Welch’s ANOVA was chosen. When one-way ANOVA showed statistically significant differences (*p* < 0.05), Games–Howell post hoc test for unequal variances or Tukey’s post hoc test for equal variances was used to check which groups differed significantly from each other. Odds ratios (OR) with 95% confidence intervals (CIs) were calculated for each postprocedural complication, comparing endoscopic with surgical drainage; a Haldane–Anscombe correction was applied in cases of zero-event cells. All reported *p*-values have been adjusted using the Bonferroni correction to account for multiple comparisons. All *p* values < 0.05 were considered statistically significant.

## 3. Results

In accordance with the STROBE recommendations, we reported the flow of patients throughout the study. During the study period, a total of 663 patients with resectable periampullary tumors were enrolled and underwent surgical resection with curative intent. Of these, eight patients who received percutaneous biliary drainage (seven PTBD and one percutaneous cholecystostomy) were excluded from the analysis due to the small sample size.

Of 655 patients, 599 patients had a PD, while 56 patients benefited from TP. The remaining 655 patients were included and stratified into three study groups: 276 patients (42.14%) without preoperative biliary drainage, 327 patients (49.92%) who underwent endoscopic drainage, and 52 patients (7.94%) who underwent surgical drainage.

A comparative analysis was conducted among patients who did not receive PBD, those who underwent endoscopic drainage, and those who underwent surgical biliary drainage. In 327 patients, endoscopic drainage was accomplished by sphincterotomy in 25 patients, PS in 250 patients, and SEMS in 36 patients. Stents of an unspecified variety were used in 16 cases for endoscopic drainage. Depending on the practitioner’s preference, different types of SEMS were inserted; seven patients had UCSEMS, twelve had PCSEMS, and one had FCSEMS. An SEMS was installed during ERBD in 16 cases, but no additional information regarding its type was provided. Surgical PBD was conducted in 52 patients prior to curative surgery, utilizing various techniques. Specifically, T-tube insertion was employed in 21 patients, cholecystostomy was employed in 13 patients, and biliodigestive diversion was employed in 18 patients. Among the biliodigestive diversion group, choledocoduodenostomy was performed in 14 cases, hepaticojejunostomy was performed in one case, and cholecystoantrostomy was performed in three cases.

### 3.1. Baseline Characteristics

We compared three groups of patients by the type of PBD method used by comparing their sex, age, primary surgical procedure, type of pancreatic anastomosis, presence of vascular invasion, type of vascular reconstruction, and the histologic type of the tumor. In the following table, different clinical variables are analyzed among patients to establish clinical homogeneity among subgroups (Table 1).

Patients with no PBD or endoscopic or surgical biliary drainage demonstrated no statistically significant difference for age, sex, primary surgical procedure, pancreatic anastomosis, vascular invasion or vascular reconstruction. However, the endoscopic group showed a higher rate of distal cholangiocarcinoma and pancreatic carcinoma compared to patients with surgical drainage or no PBD (*p* = 0.000). All the baseline characteristics are included in the following table.

### 3.2. Procedure-Related Complications

All postprocedural complication rates and mean duration of drainage are depicted in the following table (Table 2).

**Figure 1:**
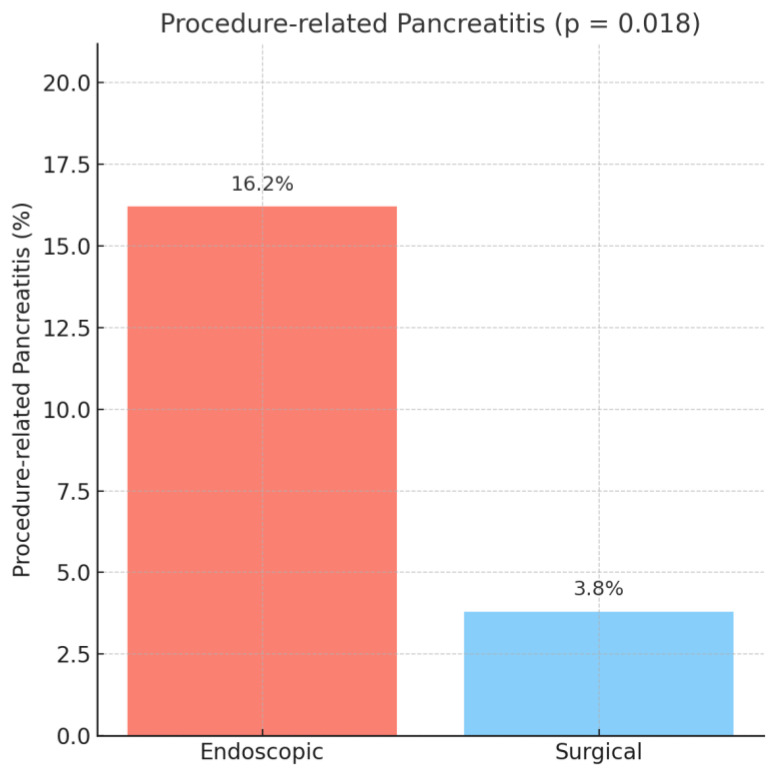
Pancreatitis rate.

In patients with preoperative biliary drainage, procedure-related pancreatitis was significantly more frequent after endoscopic drainage compared to surgical drainage (16.2% vs. 3.8%, OR = 4.84, 95% CI: 1.14–20.49, *p* = 0.018), as observed in Figure 1.

Of the 327 patients who underwent endoscopic drainage, 39 cases (11.93%) represented transient pancreatic enzyme elevations, while 14 patients (4.28%) fulfilled the formal diagnostic criteria for post-ERCP pancreatitis. In the surgical drainage group, only one patient (1.92%) developed clinically defined post-procedural pancreatitis according to the Cotton and Atlanta definitions, whereas one patient (1.92%) developed only a biological pancreatic enzyme elevation without clinical impact.

**Figure 2:**
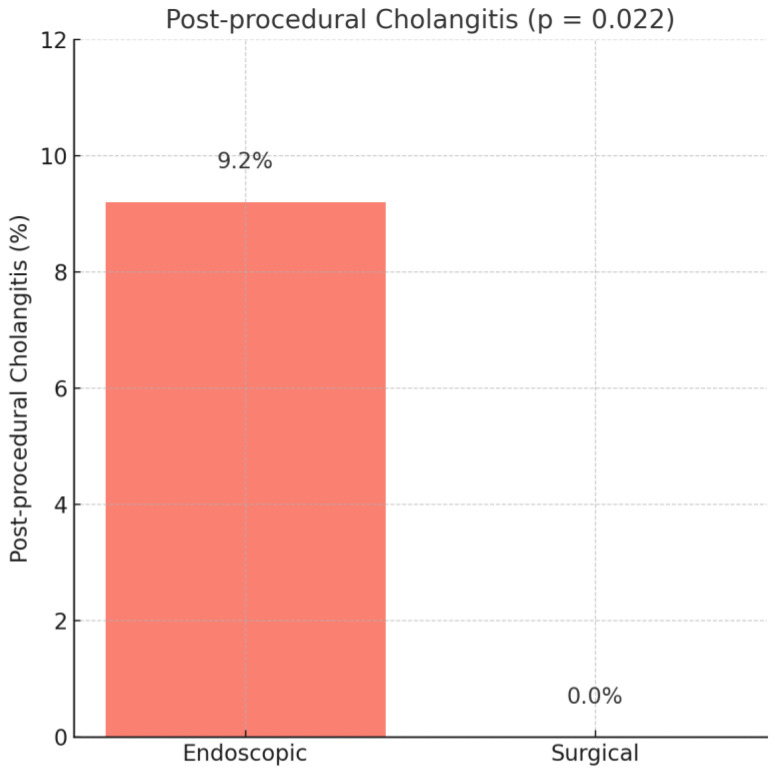
Cholangitis rate.

Cholangitis also occurred only in the endoscopic group (9.2% vs. 0%), with a markedly increased, though imprecise, odds ratio (OR = 10.76, 95% CI: 0.65–178.77, *p* = 0.022), as depicted in Figure 2.

**Figure 3:**
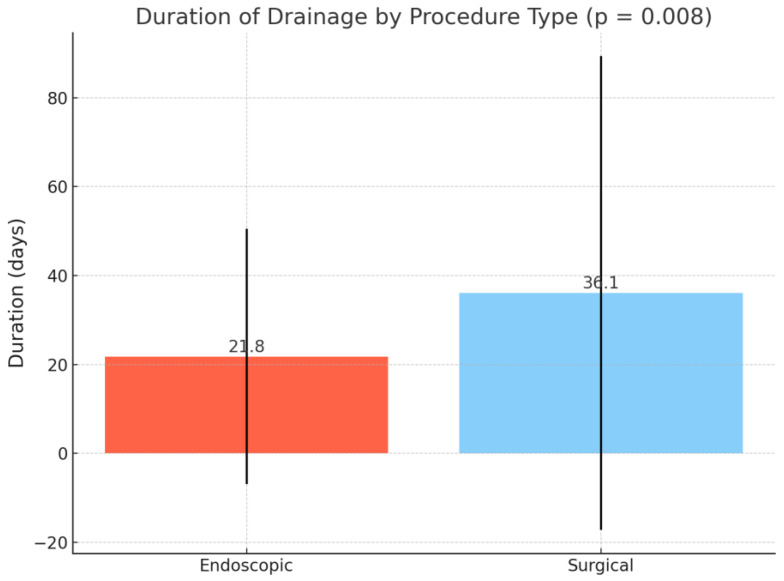
Duration of drainage (mean and standard deviation).

The rates of perforation (0.3% vs. 0%, OR = 0.48, 95% CI: 0.02–12.00, *p* = 1.000), hemorrhage (4.9% vs. 1.9%, OR = 2.62, 95% CI: 0.34–20.22, *p* = 0.487), and biliary leak (0.3% vs. 0%, OR = 0.48, 95% CI: 0.02–12.00, *p* = 1.000) were comparable between groups. The mean duration of drainage was significantly longer in the surgical group compared to the endoscopic group (36.1 ± 53.3 vs. 21.8 ± 28.7 days, *p* = 0.008) (Figure 3).

### 3.3. Postoperative Outcomes

Postoperative complication rates and postoperative prognostic indicators are graphically represented in Table 3.

**Figure 4:**
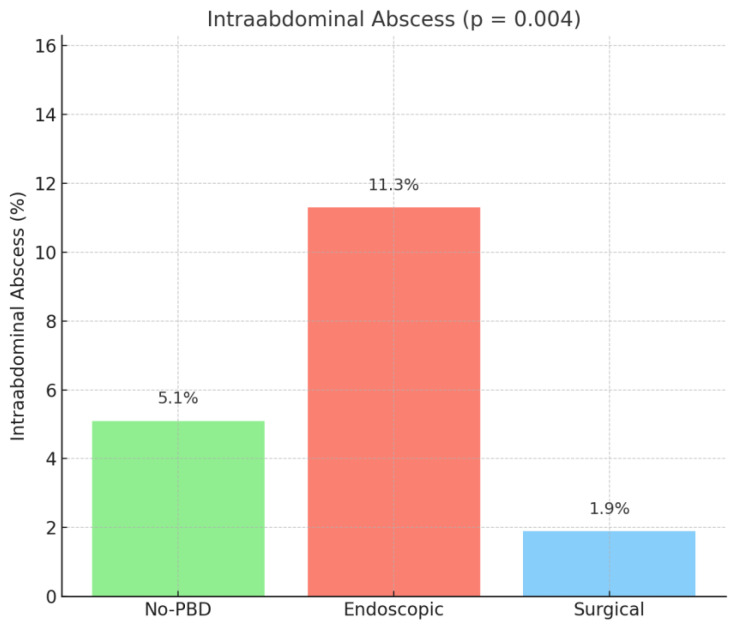
Intra-abdominal abscess rate. No PBD = no preoperative biliary drainage.

Patients with no PBD, endoscopic drainage, and surgical drainage had similar overall complication rates (129 patients (46.7%) vs. 144 patients (44%) vs. 19 (36.5%), *p* = 0.383). Wound infection had similar rates between the no-PBD, endoscopic, and surgical drainage groups (11 patients (4%) vs. 18 patients (5.5%) vs. 5 patients (9.6%), *p* = 0.214). Intra-abdominal abscess had a significantly higher occurrence in the endoscopic group (with 37 patients (11.3%)) vs. the no-PBD group (with 14 patients (5.1%)) vs. the surgical group (with 1 patient (1.9%)), with a *p* = 0.004 (Figure 4).

Similar rates of sepsis were seen among groups: 10 patients (3.6%) for no PBD, 9 patients (2.7%) for endoscopic, and 0 patients (0%) for surgical drainage (*p* = 0.537).

**Figure 5:**
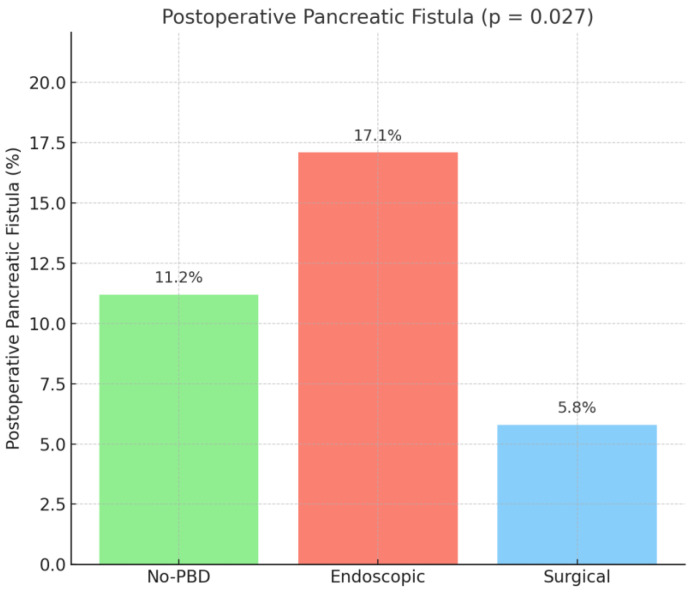
Postoperative pancreatic fistula (POPF). No PBD = no preoperative biliary drainage.

No differences were seen between groups in terms of *Clostridium difficile* infection (*p* = 0.333), pulmonary complications (*p* = 0.942), cardiovascular complications (*p* = 0.223), and acute pancreatitis (*p* = 0.155). PPH was seen in 43 patients (15.6%) from the no-PBD group, 45 patients (13.8%) from the endoscopic group, and 7 patients (13.5%) from the surgical group (*p* = 0.799). Lymph leakage had a similar occurrence among groups (*p* = 0.591). POPF had a significantly higher rate in the endoscopic group, with 56 patients (17.1%), compared to the no-PBD group with 31 patients (11.2%) and the surgical group with 3 patients (5.8%) (*p* = 0.027), with the results being displayed in Figure 5.

**Figure 6:**
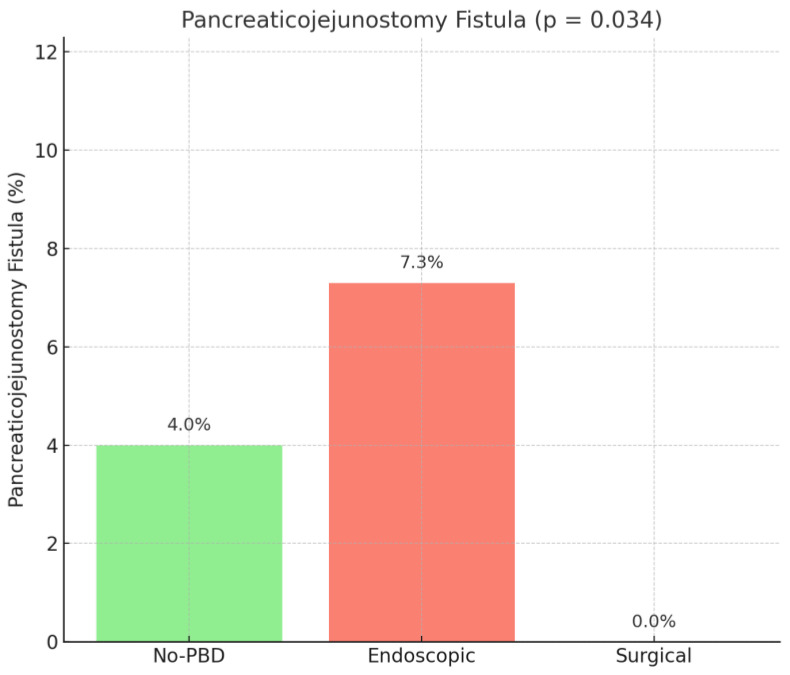
Pancreaticojejunostomy fistula rate. No PBD = no preoperative biliary drainage.

Subgroup analysis of the type of pancreatic fistula showed a higher rate of pancreaticojejunostomy fistula in the endoscopic group (*p* = 0.034), while pancreaticogastrostomy (*p* = 0.319) and pancreatic stump fistula (*p* = 0.684) had similar rates among the compared groups (Figure 6).

No statistically significant differences were determined for biliary leakage (*p* = 0.067), gastrojejunostomy leakage (*p* = 0.673), and DGE (*p* = 0.945). Moreover, vascular complications as celiac axis ischemia (*p* = 0.876) and mesenteric infarction (*p* = 0.676) did not demonstrate statistically significant differences. MOF had a similar rate among the compared groups (*p* = 0.283).

**Figure 7:**
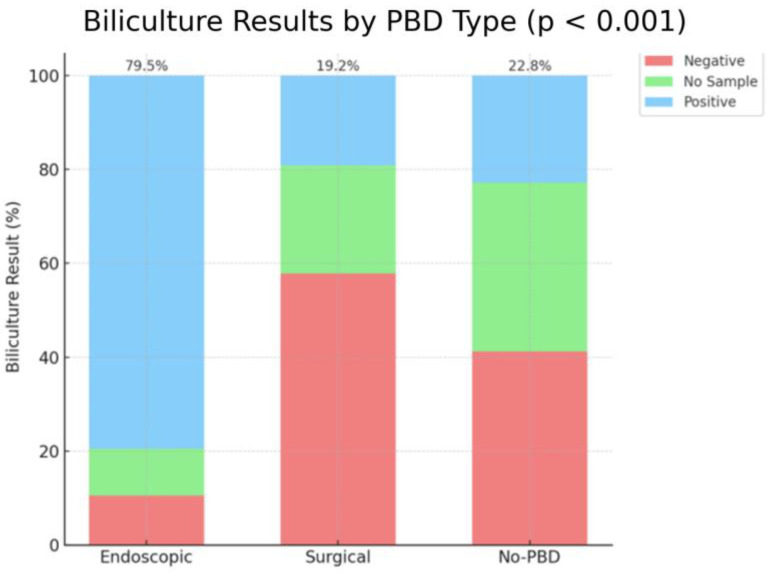
Biliculture results. No PBD = no preoperative biliary drainage.

Biliculture results had a positive result in a significantly higher percentage of patients after endoscopic drainage (79.5%) compared to the no-PBD group (22.8%) and surgical group (19.2%), with a *p*-value of *p* < 0.001 (Figure 7).

Stratification of dominant species isolated from positive bile cultures in the endoscopic versus surgical drainage groups showed *Enterococcus* spp. in 83.8% and 40% of cases, respectively, followed by *Candida* spp. (48.1% vs. 0%), *Escherichia coli* (23.1% vs. 30%), *Klebsiella* spp. (21.5% vs. 30%), *Enterobacter* spp. (7.7% vs. 20%), *Citrobacter* spp. (7.7% vs. 10%), *Pseudomonas* spp. (6.5% vs. 10%), *Proteus* spp. (1.9% vs. 10%), *Acinetobacter* spp. (2.3% vs. 10%), *Serratia* spp. (1.5% vs. 0%), and other organisms (15% vs. 10%). Given that only 10 patients in the surgical group had positive bile cultures, statistical comparison between groups was not performed, as the sample size was too limited to yield reliable results.

**Figure 8:**
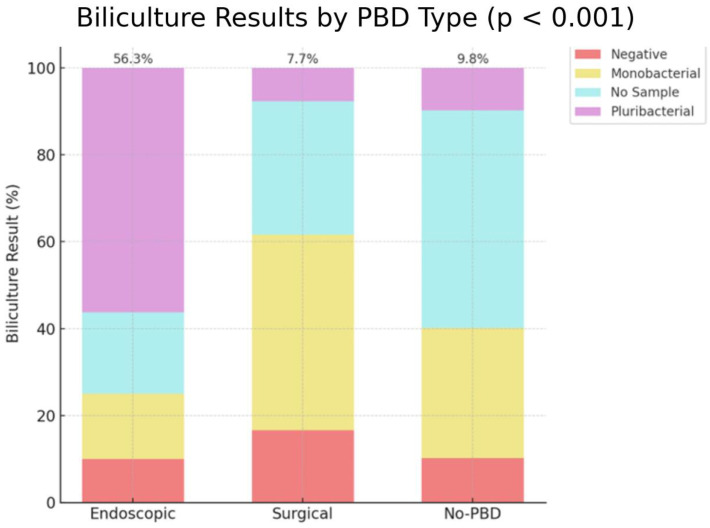
Biliculture results with monobacterial/pluribacterial results. No PBD = no preoperative biliary drainage.

Moreover, positive results of bilicultures with pluribacterial population had a higher occurrence after endoscopic drainage (56.3%) compared to no PBD (9.8%) and surgical drainage (7.7%), with a significant *p*-value < 0.001 (Figure 8).

**Figure 9:**
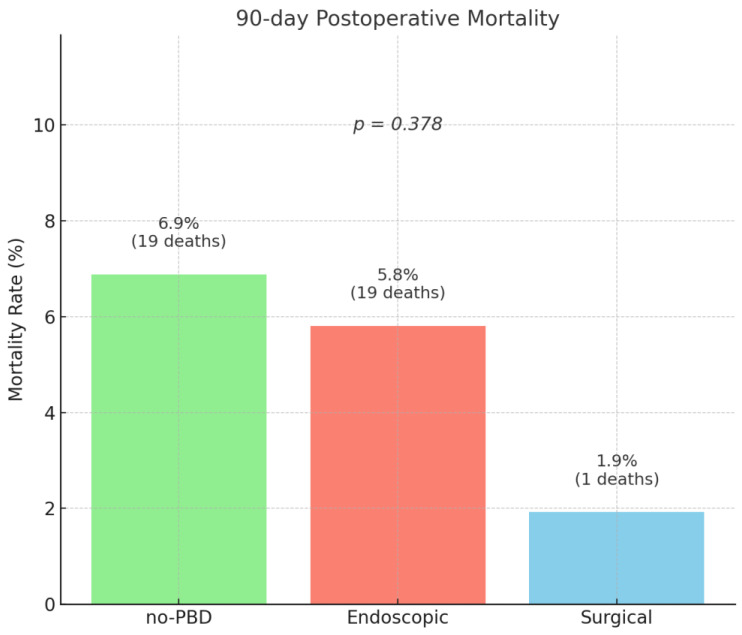
90-day postoperative mortality rate. No PBD = no preoperative biliary drainage.

Prognostic indicators such as relaparotomy rate (*p* = 0.508) and 90-day postoperative mortality (*p* = 0.378) did not show statistical differences among the compared groups (Figure 9).

## 4. Discussion

In our cohort, the choice of preoperative biliary drainage method for resectable periampullary tumors was tailored to the clinical context and institutional availability, in line with practices reported by other high-volume centers. ERBD was preferred when feasible, while surgical drainage was reserved for failed or infeasible endoscopic/percutaneous attempts, or when concomitant surgery was indicated. Conversely, in patients without established indications for drainage—such as those with moderate, asymptomatic jaundice and no cholangitis or anticipated surgical delay—PBD was not performed, consistent with current NCCN and ESMO guidelines.

Our retrospective cohort study, performed over 5 years and 5 months, demonstrated a higher rate of postoperative intra-abdominal abscess, POPF, and the POPF subgroup of pancreaticojejunostomy fistula in the endoscopic drainage group compared to the surgical drainage group and no-PBD group. Moreover, positive bilicultures, more specifically pluribacterial positive biliculture, had a significantly higher occurrence in the endoscopically stented group. Furthermore, endoscopic drainage compared to surgical drainage was associated with higher postprocedural morbidity, having a higher rate of pancreatitis and cholangitis. No significant differences were found in terms of postoperative hospital stay, relaparotomy rate, or mortality at 90 days.

Our study enhances our knowledge of surgical preoperative biliary drainage as an alternative in the case of ERCP stenting or PTBD drainage failure. Surgical biliary drainage, either by T-tube insertion, cholecystostomy, or biliodigestive diversion in the preset of a Whipple procedure, has a proven lower rate of postoperative morbidity.

However, further studies need to assess how many of the patients with surgical biliary drainage did not sustain a pancreatic resection given the postprocedural complications and mortality. This subset of patients is not analyzed by our study, as we only included the patients who managed to have surgery with curative intent for periampullary lesions.

An unexpected finding of our analysis was the lower overall complication rate associated with surgical biliary drainage compared to endoscopic drainage. This result contrasts with trends reported in several prior studies and may be partly explained by the retrospective nature of our study and potential selection bias. In our cohort, surgical drainage was typically performed in a controlled operative setting, often during planned exploratory laparotomies, whereas endoscopic drainage was more frequently performed under urgent or less-optimal clinical conditions. These contextual factors may have contributed to the observed differences in postoperative outcomes.

Additionally, the post-ERCP pancreatitis rate in our series was 16.2%, which is higher than the average rates reported by other high-volume centers. However, our observed incidence of post-ERCP pancreatitis (16.2%) was higher than the reported averages, partly because we included cases with biochemical evidence (≥ 3 × normal amylase/lipase) without clinical symptoms to ensure accurate reporting. Only 4.28% of patients had clinically defined pancreatitis according to the Cotton criteria, while 11.93% experienced what we describe as ‘pancreatic biologic reactions’. Notably, in the surgical drainage group, only one patient (1.92%) developed a clinically defined post-procedural pancreatitis, whereas one patient (1.92%) developed only a biological pancreatic enzyme elevation without clinical impact. This elevated incidence may have influenced the higher overall complication rate observed in the endoscopic group and should be considered when interpreting our results.

An additional limitation of our study is the exclusion of patients who underwent percutaneous drainage—a small but clinically relevant subgroup (n = 8). Of these, seven underwent PTBD and one underwent a percutaneous cholecystostomy. Histologically, two patients had ampullary carcinoma and six had pancreatic adenocarcinoma. Surgical procedures included seven PDs and one TP, with no vascular reconstruction required. The mean operative time was 261.8 min, with a mean estimated blood loss of 292.9 mL. The mean duration of preoperative drainage was 26.5 days. Six intraoperative bile cultures were obtained, all of which were negative. One patient developed a post-procedural bile leak requiring surgical drainage. Postoperatively, complications occurred in five of eight patients: one *Clostridium difficile* infection, one pulmonary event, one cardiovascular event, one lymphatic leak, and one PPH. No postoperative mortality was recorded, although one patient required relaparotomy for PPH. While the small sample precluded statistical comparison, presenting these data underscores the heterogeneous outcomes and highlights the continued clinical relevance of percutaneous drainage in selected scenarios.

It is important to note that the higher frequency of malignant tumors, specifically distal cholangiocarcinoma and pancreatic carcinoma, in the endoscopic group may represent a source of selection bias. This imbalance reflects real-world practice, since these malignancies are the main indication for endoscopic biliary drainage, while benign lesions rarely require preoperative decompression. Although such heterogeneity may represent a source of selection bias, it is important to note that this did not translate into significant differences in short-term mortality across groups (90-day mortality 6.9% in the no-PBD group, 5.8% in the endoscopic group, and 1.9% in the surgical drainage group, *p* = 0.378). This suggests that perioperative outcomes in our cohort were more strongly determined by drainage strategy and standardized surgical/perioperative care rather than by histological type in the immediate postoperative period. Nonetheless, the impact of tumor biology on long-term survival was beyond the scope of this study and warrants future investigation.

A further limitation of our study is the potential bias related to the heterogeneity of surgical procedures. Although the majority of patients underwent pancreaticoduodenectomy, a smaller subset had pylorus-preserving pancreaticoduodenectomy or total pancreatectomy. These procedures inherently carry different complication profiles and could therefore introduce variability in the outcomes. However, subgroup analyses revealed no significant differences in the distribution of these surgical procedures among the three study groups (no PBD, endoscopic PBD, surgical PBD), which limits the impact of this source of bias. Nevertheless, the possibility of residual confounding related to surgical technique cannot be entirely excluded.

Our findings must be interpreted in the context of the broader spectrum of PBD options available when endoscopic access is not feasible. Both surgical biliary drainage and PTBD have been described as viable alternatives, each with distinct advantages and limitations. Surgical biliary drainage offers the benefit of direct anatomical visualization and the possibility to perform concurrent bypass procedures when indicated; however, it is significantly more invasive, associated with higher perioperative morbidity, and may lead to longer hospital stays and delayed recovery [6,24]. PTBD, on the other hand, is less invasive and is particularly effective in cases of high biliary obstruction or altered anatomy. Nevertheless, it is not without drawbacks, including bile leakage, bleeding, risk of tumor seeding along the catheter tract, and patient discomfort due to prolonged external drainage [25]. Furthermore, the presence of external catheters can negatively impact quality of life and carries an additional risk of catheter dislodgement or infection [26]. The choice between these strategies should be individualized, taking into account tumor location, patient comorbidities, institutional expertise, and multidisciplinary input.

A systematic review of eight studies, performed by Lucena et al. (2018), encompassing 1116 patients, indicated that PBD leads to a higher postoperative morbidity compared to straight surgery [1]. Four studies associated PBD with a higher rate of infectious complications (wound infection, intra-abdominal abscess, and sepsis) [27,28,29,30]. Four studies demonstrated higher rates of PPH, while three studies showed more positive intraoperative bile cultures in the intervention group [28,30,31,32,33]. The control group, with no PBD, had a lower occurrence of POBF or POPF in two studies [27,33]. One of the studies demonstrated a significant increase of overall complication rate from 39% in early surgery group to 74.1% in PBD group [6]. Still, preoperative decompression of the bile duct, either endoscopic or surgical, was performed in 57.86% of all patients who underwent PD or TP.

A meta-analysis including 10,445 patients, across 27 studies included in the analysis, examined the impact of endoscopic stenting on outcomes after PD. According to the research, the ERBD group experienced considerably greater rates of wound infection, DGE, and overall morbidity than the direct surgery group. However, there was no significant difference in mortality, severe complications, POBF, POPF, PPH, or intra-abdominal abscess across the groups [34].

A Cochrane review assessed the benefits and harms of PBD versus direct surgery for obstructive jaundice, finding no significant difference in mortality but a higher rate of serious adverse events in the PBD group. The authors concluded that there is insufficient evidence to support routine PBD for obstructive jaundice and that it may increase serious adverse events [35].

The findings from a network meta-analysis suggest that, for DMBO following ERCP failure, none of the evaluated approaches (EUS-CD, EUS-HG, or surgical drainage) were superior to PTBD in terms of success rate and safety profile [36]. This is despite previous meta-analyses suggesting that EUS-BD offers higher clinical success rates and fewer complications compared to PTBD [37]. Additionally, the current analysis found no significant difference between EUS-HG and EUS-CD [36].

Another network meta-analyses based on 10 studies involving 1613 patients compared overall complication rate, wound infection, intra-abdominal infection, and PPH among no PBD, PS, SEMS, and PTBD. Wound infection was significantly more common in the SEMS group compared to no PBD, while no other statistically significant differences were found in the pairwise comparisons between groups [38].

A recent meta-analysis performed by Zhang et al. (2025) on a population of 33,516 patients who underwent a Whipple procedure were included in the study, with the aim of comparing the outcomes of PBD versus no PBD. The results showed a significantly higher rate of POPF, SSI, intra-abdominal infection, and sepsis, prolonging the hospital stay in the intervention group. On the other hand, POBF demonstrated a significantly lower rate in the patients who presented preoperative decompression of the CBD. Mortality was not significantly influenced by the preoperative approach [39].

A meta-analysis of RCTs conducted by Glazer et al. in 2014 focused on comparing endoscopic and surgical bypass for malignant bile duct obstruction. The study concluded that technical success rates were comparable (RR 0.99; 95% CI: 0.93–1.05; *p* = 0.67), with no significant differences in major complication rates (39% in surgical patients vs. 21.2% in stent patients; *p* = 0.14), 30-day mortality (15% in surgical patients vs. 12% in stent patients; *p* = 0.40), or average survival time (120 ± 37 days for surgical patients vs. 129 ± 27 days for stent patients; *p* = 0.57). However, surgical bypass demonstrated a significant advantage in reducing biliary obstruction recurrence, with a lower recurrence rate compared to stent placement (RR 0.14; 95% CI: 0.03–0.63; *p* < 0.01) [40].

Moss et al. (2006) conducted a systematic review and meta-analysis comparing endoscopic and surgical bypass for DMBO. The study concluded that PS placement, compared to surgical drainage—primarily biliodigestive diversion—was associated with a lower risk of complications (RR 0.60; 95% CI: 0.45–0.81; *p* < 0.001) but a significantly higher risk of recurrent biliary obstruction (RR 18.59; 95% CI: 5.33–64.86; *p* < 0.001). No statistically significant difference was observed in 30-day mortality between the two groups (RR 0.58; 95% CI: 0.32–1.04; *p* = 0.07) [41].

An important area of interest in preoperative biliary drainage is the influence on intraoperative biliculture results. Various studies comparing endoscopic stenting with other PBD methods demonstrated a significantly higher percentage of positive bilicultures, especially pluribacterial population, in the endoscopic group [3,31,42,43,44,45]

The association between positive bile cultures and an increased risk of postoperative infectious complications—such as wound infection, intra-abdominal abscess, sepsis, POPF, and POBF—is already well documented in the literature [31,42,43]. Therefore, we did not conduct a detailed microbiological analysis of each isolate, as it was beyond the scope of our study. Our findings on the significantly higher rate of positive and multibacterial bile cultures in the endoscopic group provide context for the observed differences in postoperative infectious complication rates among the groups compared.

Our findings on the significantly higher rate of positive and multibacterial bile cultures in the endoscopic group align with existing literature, supporting the notion that preoperative endoscopic drainage predisposes to bile contamination, which may subsequently increase infectious morbidity.

Although detailed bacterial profiles and resistance patterns were not analyzed, we deliberately included bile culture positivity and multibacterial contamination rates as key variables, given their known clinical relevance in the postoperative course.

These results have potential clinical implications. While endoscopic drainage remains the first-line option due to its minimally invasive nature, surgical drainage should not be viewed solely as a salvage approach but as a viable alternative in patients with failed or contraindicated ERCP, especially in high-volume centers. By linking our data with international evidence and guidelines (ESGE, ASGE, NCCN and ESMO), we emphasize that patient selection, multidisciplinary assessment, and local expertise are critical for optimizing perioperative outcomes.

Therapeutic EUS has emerged as a pivotal tool in the management of complications associated with periampullary tumors, particularly in cases of double obstruction involving both the biliary tract and gastric outlet. In this setting, EUS-BD and EUS-GE provide effective, minimally invasive alternatives when conventional endoscopic or percutaneous approaches are not feasible or have failed. EUS-BD techniques are most often applied with palliative intent, providing minimally invasive alternatives in patients who are not candidates for definitive surgical resection. In the context of resectable periampullary tumors, EUS-BD may be considered a preoperative option when ERCP is unsuccessful or not feasible; however, it has not yet been established as a standard approach for upfront biliary drainage before curative surgery. The growing evidence supporting the safety and efficacy of therapeutic EUS underscores its expanding role in the multidisciplinary management of periampullary cancers [46].

The strengths of our study include the large sample size of 655 patients, all treated in a high-volume pancreatic surgery center with standardized protocols, and the direct head-to-head comparison of three clinically relevant strategies (no PBD, endoscopic drainage, and surgical drainage). Furthermore, the study provides one of the largest single-center European cohorts addressing this topic, enhancing the reliability of our findings and contributing novel evidence of the role of surgical drainage as an alternative to endoscopic stenting.

However, the conflicting results emphasize the need for further research, particularly RCTs, to evaluate alternative PBD methods and generate higher-quality evidence to inform future guidelines.

## 5. Conclusions

This large retrospective cohort study investigated the impact of different PBD strategies—endoscopic, surgical, or none—on perioperative outcomes in patients undergoing pancreatic surgery for resectable periampullary tumors. Our findings highlight that surgical biliary drainage is associated with significantly lower perioperative morbidity compared to endoscopic drainage. Specifically, patients in the endoscopic group experienced higher rates of POPF, pancreaticojejunostomy fistula, and intra-abdominal abscesses, along with significantly more frequent positive bile cultures—particularly pluribacterial ones. Moreover, the endoscopic approach showed a higher incidence of procedure-related complications such as post-ERCP pancreatitis and cholangitis.

Importantly, despite these differences in complication profiles, the overall postoperative hospital stay, need for relaparotomy, and 90-day mortality rates were comparable across groups, suggesting that surgical drainage can achieve equivalent long-term safety while reducing specific postoperative morbidities.

While endoscopic biliary drainage remains the most widely used method due to its minimally invasive nature and accessibility, our results suggest that surgical drainage may be a valuable and potentially safer alternative in selected cases—particularly in patients with failed or contraindicated ERCP, or when additional surgical procedures are planned.

These findings underscore the importance of individualized decision-making based on patient-specific factors, institutional expertise, and multidisciplinary evaluation. They also support a more nuanced approach to preoperative biliary drainage that considers surgical drainage not merely as a salvage technique but as a potentially preferable first-line strategy in appropriate settings.

Given the retrospective nature of our study and its inherent limitations—including selection bias, the exclusion of PTBD cases due to small sample size, and an analysis limited to patients undergoing curative resection—our conclusions warrant cautious interpretation. Future prospective, randomized controlled trials are needed to validate these findings and to clarify the optimal indications and timing for each PBD strategy. Such data will be instrumental in shaping evidence-based guidelines for the perioperative management of patients with periampullary neoplasms.

## 6. Limitations

Several limitations should be acknowledged when interpreting the findings of this retrospective cohort study. First, the retrospective, non-randomized design inherently introduces the potential for selection bias, residual confounding, and information bias. The allocation to different PBD strategies was not randomized but based on multidisciplinary and surgeon-led clinical decision-making, which may have been influenced by patient comorbidities, anatomical complexity, or logistical factors. These uncontrolled variables could affect the internal validity of comparative analyzes.

Second, although we compared three groups (no PBD, endoscopic PBD, and surgical PBD), patients who underwent PTBD were excluded from the analysis due to their small number (n = 8). This limits the comprehensiveness of our assessment, as PTBD is an important alternative in routine clinical practice and especially relevant in patients with complex biliary anatomy or failed ERCP. The exclusion of this group precludes meaningful conclusions regarding its comparative safety and efficacy.

Third, the analysis included only patients who ultimately underwent surgical resection with curative intent. Patients who received PBD but were subsequently deemed unresectable, either due to disease progression or drainage-related complications, were not included. This introduces a potential survivorship bias and may underestimate the true morbidity associated with each drainage method, particularly for procedures performed in high-risk patients.

Fourth, some data elements, such as the timing between drainage and surgery, detailed technical characteristics of the endoscopic or surgical interventions, and severity grading of complications, were not uniformly available across all cases. As such, we were unable to control for potential confounding related to procedural nuances (e.g., type and duration of stent placement, laparoscopic vs. open drainage) that may influence outcomes.

Fifth, microbiological assessment was limited to biliculture results, without evaluating antimicrobial resistance patterns or associations with postoperative infectious complications in detail. Moreover, the high rate of positive bile cultures in the endoscopic group may reflect longer indwelling stent duration or more frequent interventions, but these variables were not fully explored in stratified analysis.

Sixth, a multivariate regression analysis was not performed, mainly due to the retrospective design and the small size of the surgical drainage cohort; instead, we relied on subgroup comparisons and baseline characteristic analyses to ensure group comparability.

Finally, the single-center nature of the study, although advantageous for procedural consistency and data homogeneity, may limit the external validity and generalizability of our findings to institutions with differing expertise, case volumes, or perioperative protocols.

In light of these limitations, our findings should be interpreted with caution and regarded as hypothesis-generating. Prospective, multicenter RCTs are warranted to establish causal relationships and guide the optimal selection of preoperative biliary drainage strategies in patients with periampullary tumors.

## Figures and Tables

**Table 1:** Baseline characteristics.

Variable	All Patients (n = 655)	No PBD (n = 276)	Endoscopic (n = 327)	Surgical (n = 52)	Pooled *p*-Value
Age, mean (SD)	62.3 (10.4)		64.1 (10)	64.5 (7.2)	0.059
Sex					0.263
Female	273 (41.7%)	124 (45.4%)	126 (46.1%)	23 (8.4%)	
Male	382 (58.3%)	152 (39.8%)	201 (52.6%)	29 (7.6%)	
Histology of lesion					0.000
Ampullary carcinoma	127 (19.4%)	54 (42.5%)	65 (51.2%)	8 (6.3%)	
Benign lesions	23 (3.5%)	16 (69.6%)	7 (30.4%)	0 (0%)	
Chronic pancreatitis	21 (3.2%)	17 (80.9%)	4 (19.0%)	0 (0%)	
Distal cholangiocarcinoma	66 (10.1%)	17 (25.7%)	42 (63.6%)	7 (10.6%)	
Duodenal carcinoma	12 (1.8%)	10 (83.3%)	1 (8.3%)	1 (8.3%)	
GIST	8 (1.2%)	8 (100%)	0 (0%)	0 (0%)	
NET	25 (3.8%)	19 (76%)	6 (24%)	0 (0%)	
Other carcinoma sites	4 (0.6%)	4 (100%)	0 (0%)	0 (0%)	
Pancreatic carcinoma	354 (54%)	118 (33.3%)	200 (56.5%)	36 (10.2%)	
Premalignant lesions	15 (2.3%)	13 (86.7%)	2 (13.3%)	0 (0%)	
Primary surgical procedure					0.223
PD	564 (86.1%)	230 (40.8%)	286 (50.7%)	48 (8.5%)	
PPPD	35 (5.3%)	16 (45.7%)	18 (51.4%)	1 (2.8%)	
PPTP	12 (1.8%)	5 (41.7%)	7 (58.3%)	0 (0%)	
TP	44 (6.7%)	25 (56.8%)	16 (36.3%)	3 (6.8%)	
Pancreatic anastomosis					0.498
PG	410 (62.6%)	174 (42.4%)	204 (49.7%)	32 (7.8%)	
PJ	187 (28.5%)	70 (37.4%)	100 (53.5%)	17 (9.1%)	
Vascular invasion					0.185
IVC	2	0 (0%)	2 (100%)	0 (0%)	
SMV	22	7 (31.8%)	11 (50%)	4 (18.2%)	
PV	22	6 (27.27%)	15 (68.2%)	1 (4.5%)	
PV + SMV	3	0 (0%)	3 (100%)	0 (0%)	
SV	1	1 (100%)	0 (0%)	0 (0%)	
no	605	262 (43.3%)	296 (48.9%)	47 (7.8%)	
Vascular reconstruction					0.197
Tangential suture	42	13 (30.9%)	24 (57.1%)	5 (11.9%)	
T-T anastomosis	6	0 (0%)	6 (100%)	0 (0%)	

GIST: gastrointestinal stromal tumor; IVC: inferior vena cava; NET: neuroendocrine tumor; PBD: preoperative biliary drainage; PD: pancreaticoduodenectomy; PG: pancreaticogastrostomy; PJ: pancreaticojejunostomy; PPPD: pylorus-preserving pancreaticoduodenectomy; PPTP: pylorus-preserving total pancreatectomy; PV: portal vein; PV-SMV: portal vein-superior mesenteric vein confluence; SD: standard deviation; SMV: superior mesenteric vein; SV: splenic vein; TP: total pancreatectomy; T-T: termino-terminal.

**Table 2:** Postprocedural complication rates.

Variable	All Patients with PBD (n = 379)	Endoscopic (n = 327)	Surgical (n = 52)	Pooled *p*-Value
Pancreatitis	55 (14.5%)	53 (16.2%)	2 (3.8%)	0.018
Cholangitis	30 (7.9%)	30 (9.2%)	0 (0%)	0.022
Perforation	1 (0.3%)	1 (0.3%)	0 (0%)	1
Hemorrhage	17 (4.5%)	16 (4.9%)	1 (1.9%)	0.487
Biliary leak	1 (0.3%)	1 (0.3%)	0 (0%)	1
Duration of drainage, mean (SD)	23.80 ± 33.45	21.84 ± 28.73	36.12 ± 53.25	0.008

PBD: preoperative biliary drainage; SD: standard deviation.

**Table 3:** Postoperative complication rates.

Variable	All Patients (n = 655)	No PBD (n = 276)	Endoscopic (n = 327)	Surgical (n = 52)	Pooled *p*-Value
Overall complication rate	292 (44.6%)	129 (46.7%)	144 (44.0%)	19 (36.5%)	0.383
Wound infection	34 (5.2%)	11 (4.0%)	18 (5.5%)	5 (9.6%)	0.214
Intra-abdominal abscess	52 (7.9%)	14 (5.1%)	37 (11.3%)	1 (1.9%)	0.004
Sepsis	19 (2.9%)	10 (3.6%)	9 (2.7%)	0 (0%)	0.537
*Clostridium difficile* colitis	42 (6.4%)	21 (7.6%)	20 (6.1%)	1 (1.9%)	0.333
Pulmonary complications	44 (6.7%)	20 (7.2%)	21 (6.4%)	3 (5.8%)	0.942
Cardiovascular complications	38 (5.8%)	21 (7.6%)	14 (4.3%)	3 (5.8%)	0.223
Acute pancreatitis	25 (3.8%)	15 (5.4%)	8 (2.4%)	2 (3.8%)	0.155
Post-pancreatectomy hemorrhage (PPH)	95 (14.5%)	43 (15.6%)	45 (13.8%)	7 (13.5%)	0.799
Lymph leakage	12 (1.8%)	4 (1.4%)	8 (2.4%)	0 (0%)	0.591
POPF	90 (13.7%)	31 (11.2%)	56 (17.1%)	3 (5.8%)	0.027
Pancreaticojejunostomy fistula	35 (5.3%)	11 (4%)	24 (7.3%)	0 (0%)	0.034
Pancreaticogastrostomy fistula	52 (7.9%)	18 (6.5%)	31 (9.5%)	3 (5.8%)	0.319
Pancreatic stump fistula	3 (0.4%)	2 (0.7%)	1 (0.3%)	0 (0%)	0.684
Biliary leakage	28 (4.3%)	17 (6.1%)	11 (3.4%)	0 (0%)	0.067
Gastrojejunostomy leakage	9 (1.4%)	3 (1.1%)	6 (1.8%)	0 (0%)	0.673
Delayed gastric emptying (DGE)	46 (7.0%)	19 (6.9%)	23 (7.0%)	4 (7.7%)	0.945
Celiac axis ischemia	9 (1.4%)	5 (1.8%)	4 (1.2%)	0 (0%)	0.876
Mesenteric infarction	23 (3.5%)	12 (4.3%)	10 (3.0%)	1 (1.9%)	0.676
Multiple organ failure (MOF)	66 (10.1%)	31 (11.2%)	33 (10.1%)	2 (3.8%)	0.283
Relaparotomy	49 (7.5%)	20 (7.2%)	23 (7.0%)	6 (11.5%)	0.508
Postoperative hospital stay, mean (SD)	15.19 ± 11.03	15.99 ± 11.05	14.46 ± 9.67	15.50 ± 17.16	0.241
90-day mortality	39 (5.9%)	19 (6.9%)	19 (5.8%)	1 (1.9%)	0.378

PBD: preoperative biliary drainage; POPF: postoperative pancreatic fistula; SD: standard deviation.

## Data Availability

All data were obtained from the databases. The author has sorted out all the data and attached them to the attachment at the end of the article.

## Supplementary Materials

The following supporting information can be downloaded at: https://www.mdpi.com/article/10.3390/medicina61091565/s1, STROBE Statement—Checklist of items that should be included in reports of cohort studies. Supplementary Table S1. Postprocedural Complications (Endoscopic vs Surgical drainage). Supplementary Table S2. Odds Ratios (OR) with 95% Confidence Intervals (CI) and *p*-values for Postoperative Complications.

## Abbreviations

The following abbreviations are used in this manuscript:

ASGE	American Society for Gastrointestinal Endoscopy
CBD	Common bile duct
DGE	Delayed gastric emptying
DMBO	Distal malignant biliary obstruction
ERBD	Endoscopic retrograde biliary drainage
ERCP	Endoscopic retrograde cholangiopancreatography
ESGE	European Society of Gastrointestinal Endoscopy
ESMO	European Society for Medical Oncology
EUS	Endoscopic ultrasound
EUS-BD	Endoscopic ultrasound-guided biliary drainage
EUS-CD	EUS-guided choledochoduodenostomy
EUS-GE	Endoscopic ultrasound-guided gastroenterostomy
EUS-HG	EUS-guided hepaticogastrostomy
FCSEMS	Fully covered self-expandable metallic stent
GIST	Gastrointestinal stromal tumor
IVC	Inferior vena cava
ISGLS	International Study Group of Liver Surgery
ISGPS	International Study Group of Pancreatic Surgery
MBD	Main biliary duct
MOF	Multiple organ failure
NCCN	National Comprehensive Cancer Network
NET	Neuroendocrine tumor
PBD	Preoperative biliary drainage
PCSEMS	Partially covered self-expandable metallic stent
PD	Pancreaticoduodenectomy
PG	Pancreaticogastrostomy
PJ	Pancreaticojejunostomy
POBF	Postoperative biliary fistula
POPF	Postoperative pancreatic fistula
PPH	Post-pancreatectomy hemorrhage
PPPD	Pylorus-preserving pancreaticoduodenectomy
PPTP	Pylorus-preserving total pancreatectomy
PS	Plastic stent
PTBD	Percutaneous transhepatic biliary drainage
PV	Portal vein
PV-SMV	Portal vein-superior mesenteric vein confluence
RCT	Randomized controlled trial
SEMS	Self-expandable metallic stent
SD	Standard deviation
SMV	Superior mesenteric vein
SSI	Surgical site infection
STROBE	STrengthening the Reporting of OBservational studies in Epidemiology
SV	Splenic vein
TP	Total pancreatectomy
T-T	Termino-terminal
UCSEMS	Uncovered self-expandable metallic stent
