# Supplementary material for: Biliary Drainage for the Preoperative Management of Periampullary Neoplasms: A Retrospective Cohort Study
Source: Medicina (Kaunas). 2025 Aug 30;61(9):1565. doi: 10.3390/medicina61091565 (PMC12471752; doi:10.3390/medicina61091565)
Supplement: Supplementary file 1 [file medicina-61-01565-s001.zip › medicina-3794054-Supplementary_Tables.pdf]

Supplementary Table S1. Postprocedural Complications (Endoscopic vs Surgical drainage)

Complication	Endoscopic (n=327)	Surgical (n=52)	Odds Ratio (95% CI)	p-value
Pancreatitis	53 (16.2%)	2 (3.8%)	4.84 (1.14–20.49)	0.018
Cholangitis	30 (9.2%)	0 (0%)	10.76 (0.65–178.77)	0.022
Perforation	1 (0.3%)	0 (0%)	0.48 (0.02–12.00)	1.000
Hemorrhage	16 (4.9%)	1 (1.9%)	2.62 (0.34–20.22)	0.487
Biliary leak	1 (0.3%)	0 (0%)	0.48 (0.02–12.00)	1.000
Duration of drainage (days, mean ± SD)	21.84 ± 28.73	36.12 ± 53.25	—	0.008

Supplementary Table S2. Odds Ratios (OR) with 95% Confidence Intervals (CI) and p-values for Postoperative Complications

Complication	Endoscopic vs No-PBD	Surgical vs No-PBD	Endoscopic vs Surgical	p-value
Overall complication rate	0.90 (0.65-1.24)	0.66 (0.36-1.21)	1.37 (0.75-2.50)	0.383
Wound infection	1.40 (0.65-3.02)	2.56 (0.85-7.71)	0.55 (0.19-1.54)	0.214
Intra-abdominal abscess	2.39 (1.26-4.52)	0.37 (0.05-2.85)	6.51 (0.87-48.49)	0.004
Sepsis	0.75 (0.30-1.88)	0.24 (0.01-4.19)	3.13 (0.18-54.62)	0.537
Clostridium difficile colitis	0.79 (0.42-1.49)	0.24 (0.03-1.81)	3.32 (0.44-25.30)	0.333

Pulmonary complications	0.88 (0.47-1.66)	0.78 (0.22-2.74)	1.12 (0.32-3.90)	0.942
Cardiovascular complications	0.54 (0.27-1.09)	0.74 (0.21-2.59)	0.73 (0.20-2.63)	0.223
Acute pancreatitis	0.44 (0.18-1.05)	0.70 (0.15-3.14)	0.63 (0.13-3.04)	0.155
Post-pancreatectomy hemorrhage (PPH)	0.86 (0.55-1.36)	0.84 (0.36-1.99)	1.03 (0.44-2.42)	0.799
Lymph leakage	1.71 (0.51-5.73)	0.58 (0.03-10.87)	2.79 (0.16-49.13)	0.591
POPF	1.63 (1.02-2.62)	0.48 (0.14-1.65)	3.38 (1.02-11.21)	0.027
Pancreaticojejunostomy fistula	1.91 (0.92-3.97)	0.22 (0.01-3.79)	8.48 (0.51-141.53)	0.034
Pancreaticogastrostomy fistula	1.50 (0.82-2.75)	0.88 (0.25-3.09)	1.71 (0.50-5.81)	0.319
Pancreatic stump fistula	0.42 (0.04-4.66)	1.05 (0.05-22.10)	0.48 (0.02-12.00)	0.684
Biliary leakage	0.53 (0.24-1.15)	0.14 (0.01-2.39)	3.82 (0.22-65.73)	0.067
Gastrojejunostomy leakage	1.70 (0.42-6.87)	0.74 (0.04-14.62)	2.12 (0.12-38.25)	0.673
Delayed gastric emptying (DGE)	1.02 (0.55-1.92)	1.13 (0.37-3.46)	0.91 (0.30-2.74)	0.945
Celiac axis ischemia	0.67 (0.18-2.52)	0.47 (0.03-8.63)	1.46 (0.08-27.53)	0.876
Mesenteric infarction	0.69 (0.30-1.63)	0.43 (0.05-3.39)	1.61 (0.20-12.84)	0.676
Multiple organ failure (MOF)	0.89 (0.53-1.49)	0.32 (0.07-1.36)	2.81 (0.65-12.06)	0.283
Relaparotomy	0.97 (0.52-1.80)	1.67 (0.64-4.38)	0.58 (0.22-1.50)	0.508

90-day mortality	0.83 (0.43-1.61)	0.27 (0.03-2.03)	3.15 (0.41-24.02)	0.378
